# Evaluation of Outcome and Tolerability of Combination Chemotherapy with Capecitabine and Oxaliplatin as First Line Therapy in Advanced Gastric Cancer

**Published:** 2016-10-01

**Authors:** Mohammad Ali Mashhadi, Zahra Sepehri, Ali Reza Bakhshipour, Ali Zivari, Hossein Ali Danesh, Hasan Ali Metanat, Azra Karimkoshteh, Seyed Mehdi Hashemi, Hossein Rahimi, Zohre Kiani

**Affiliations:** 1 Professor, Health Promotion Research Center, Zahedan University of Medical Sciences, Zahedan, Iran; 2 Assistant Professor of Internal Medicine, Department of Internal Medicine, Amir Al-Momenin Hospital, Zabol University of Medical Sciences, Zabol, Iran; 3 Associate Professor, Department of Internal Medicine, Ali-Ebne Abitaleb Hospital, Zahedan University of Medical Sciences, Zahedan, Iran; 4 Assistant Professor, Department of Internal Medicine, Ali-Ebne Abitaleb Hospital, Zahedan University of Medical Sciences, Zahedan, Iran; 5 Assistant Professor of Surgical Medicine, Department of Surgical Medicine, Ali-Ebne Abitaleb Hospital, Zahedan University of Medical Sciences, Zahedan, Iran; 6 Assistant Professor of Internal Medicine, Department of Internal Medicine, Ghaem Hospital, Mashhad University of Medical Sciences, Mashhad, Iran; 7 Student Research Committee, Zabol University of Medical Sciences, Zabol, Iran; 8 Student Research Committee, Kerman University of Medical Sciences, Kerman, Iran

**Keywords:** Advanced gastric cancer, Capecitabine, Oxaliplatin

## Abstract

**Background:** Combination chemotherapy is accepted as a high efficacy treatment for gastric cancer, whereas choice of standard treatment is unclear. Multiple chemotherapeutic regimens have been used to achieve higher efficacy and lower toxicity. This study was designed to evaluate the treatment results of advanced gastric cancer with Capecitabine and Oxaliplatin regimen.

**Subjects and Methods** **:** All cases with documented gastric adenocarcinoma and advanced disease were candidates for receiving Xelox regimen (Capecitabine – 750 mg/m^2^/twice daily/ 1-14 days and Oxaliplatin 125 mg/m^2^ in 1st day).

**Results:** Twenty five cases with advanced gastric cancer entered in study while 24 cases continued treatment protocol and were evaluated. Mean age was 59.5 ± 12.1 years (range: 20-75), male and female cases were 66.7% and 33.3%, respectively. All cases received at least four cycles of Xelox regimen. Overall response rate was 74.99% with 29.16% complete response. Overall survival rate was 13 ± 0.53 months and DFS (disease-free survival) was 6 ± 1.09 months. Extremities neuropathy (62.5%), headache (45.8%) and muscle cramps (29.2%) were the most common complains. Haematological changes were rare and 16.7% of cases had mild cytopenia. Treatment related death was not observed.

**Conclusion:** Xelox regimen is a safe and highly effective first line treatment for gastric cancer; however, considering it as first line therapy needs larger studies.

## Introduction

 Gastric cancer is one of the five most common types of cancers in the world and second most common cause of cancer-related death in the world.^1^^-^^4^ Improvement in diagnosis and treatment of gastric cancer is a clear finding in recent years; however, many cases present with advanced disease. This leads to poor prognosis and switching treatment protocol to palliative chemotherapy. Hence, researches are in progress for more effective treatments.^5^^-^^8^ There is no standard treatment for advanced gastric cancer and classic regimen with cisplatin, fluorouracil and epirubicine lead to response rate of 20-40%.^9^ This classic regimen had large toxicity and was poorly tolerated.^10^^,^^11^ Capecitabine (Xeloda®; Hoffmann-La Roche Switzerland) had potential effects of 5-FU with better tolerance rate and oral consumption.^12^^,^^13^ This drug is active in advanced gastric cancer with response rate of 28% in literatures.^14^ In combination with other drugs as first line therapy such as cisplatin, oxaliplatin, epirubicin and docetaxel, it had a response rate of 40-68%.^15^^-^^17^ Oxaliplatin is an alkylating agent with better effect on inhibition of DNA synthesis than cisplatin.^18^ Toxicity of oxaliplatin is less than cisplatin.^19^ In literature review, it has been reported that combination of capecitabine and oxaliplatin (Xelox) is more effective in treatment of advanced gastric cancer.^20^^-^^22^ The aim of this study was to evaluate treatment results of advanced gastric cancer with capecitabine and oxaliplatin.

## SUBJECTS AND METHODS

**Patient selection**

Patients with documented unresectable gastric adenocarcinoma (confirmed with spiral CT scan) or metastatic disease were registered and entered the study. This study was approved by ethical committee of Zahedan University of Medical Sciences and consent forms were filled by all participants in the study (Ethical code number: zaums.1.REC.1391.950). Inclusion criteria were: age>18 years, performance status>70 according to karnofsky score, life expectancy>4 months, Hb>9 gr/dl, normal liver and kidney and heart function tests. Exclusion criteria were: non cooperative patients, performance status<70, abnormal vital organ function tests, brain metastasis, bone marrow involvement, previous treatment.

**Treatment protocol**

All eligible cases received at least 3 cycles (3-8 cycles) of Xelox regimen; oxaliplatin 125 mg/m^2^ in day 1 and capecitabine 750 mg/m^2^ BID for 2 weeks and 1 week rest and then other course of treatment protocol was repeated. If the patients could not receive at least 3 cycles of Xelox regimen, or showed disease progression in the first 3 cycles of treatment protocol, or were non cooperative for continuing oral drug administration or severe experienced treatment-related toxicities due to oxaliplatine, the treatment protocol would changed. Patients' evaluations prior to start of treatment were: careful physical examination, performance status estimation, Complete Blood Count (CBC), biochemistry, chest X-Ray. During treatment, all parameters were checked prior to beginning of next courses of treatment. Spiral abdominal CT scan was done at baseline and after every three courses of Xelox regimen to evaluate tumor response. Tumor response was evaluated according to RECIST guidelines. Treatment-related toxicities were reported according to National Cancer Institute Common Toxicity Criteria (NCI-CTC) version 2.0 toxicity scale.

## Results

 Over three years (2011-2013), 24 cases continued treatment protocol and were evaluated out of 25 eligible cases. Male and female cases were 66.7% and 33.3% of all, respectively. Mean age was 59.5 ± 12.1 years (ranged between 20 and 75). All cases received at least four courses of Xelox regimen. Patients' characteristics are summarized in Table 1.

Response rates in male and female are summarized in Table 2 and 3. Overall response rate was 74.99%. Seven cases (29.16%) had complete response.

**Table 1:** Baseline patients' characteristics

**Parameters**	**Number (%)**
Sex Male Female	16 (66.7) 8 (33.3)
Site of metastasis Liver Lung Peritoneum Lymph node	8 (33.33) 1 (4.16) 4 (16.66) 11 (45.83)
Performance status 100 90 80 70	7 (29.16) 8 (33.33) 6 (25) 3 (12.5)

**Table 2:** Responses in patients based on their sex

	**With response**	**Without response**	**p-value**
**Male**	12 (75%)	4 (25%)	0.99
**Female**	6 (75%)	2 (25%)	
**Total**	18 (75%)	6 (25%)	

**Figure 1:**
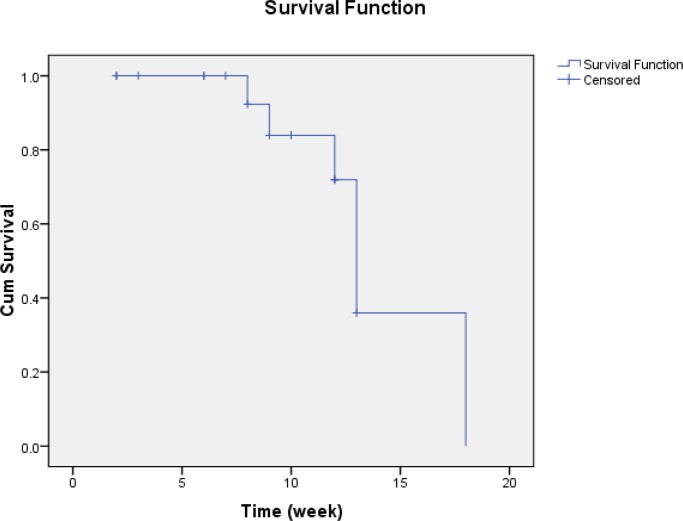
The overall survival rate for patients

**Figure 2:**
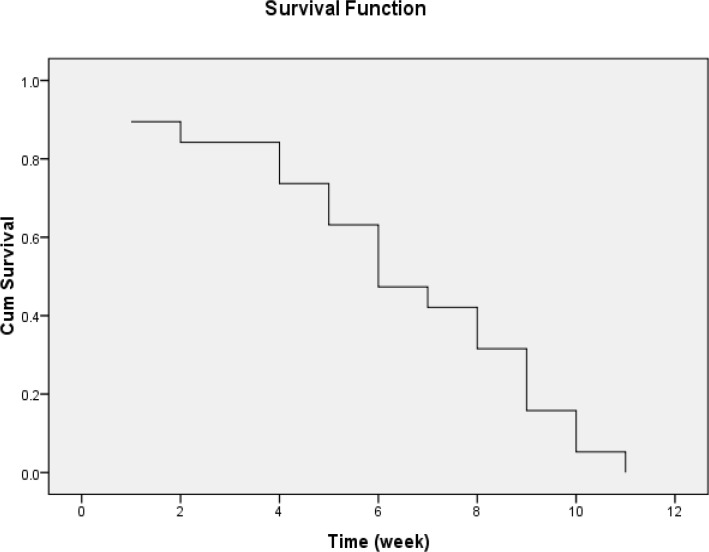
Disease free survival

We observed partial response in 11 cases (45.83%), progressive disease in three cases (12.5%) and no response with stable disease in three cases (12.5%). Overall survival rate was 13 ± 0.53 months (Figure 1) and disease-free survival (DFS) was 6 ± 1.09 months (Figure 2). All cases were evaluated for treatment-related toxicities. The most common toxicities were neuropathy, headache, dizziness and muscle cramp (Table 4). Our results showed that all toxicities were in grade 1 and 2; while we did not observe any toxicity in grade 3 and 4. For this reason, we did not consider dose modification in current study. Overall response rate was 74.99% and near to 30% of cases had complete response.

**Table 3:** Responses characteristic according Mann-Whitney test

**Response**	**Number (%)**	**Mean ± SD**	**p-value**
**With response**	18 (75)	59.4 ± 13.6	0.923
**Without response**	6 (25)	40.6 ± 6.7	

**Table 4:** Treatment related toxicities

**Toxicities**	**Number (%)**
Extremity neuropathy	15 (62.5)
Headache and dizziness	11 (45.8)
Muscle cramp	7 (29.2)
Cytopenia	4 (16.7)
Hand foot syndrome	2 (8.4)
Diarrhoea	1 (4.2)
Nausea and vomiting	1 (4.2)
Abdominal pain	1 (4.2)
Hearing loss	1 (4.2)
Anaemia	1 (4.2)
Without side effect	6 (25.2)

## Discussion

 In this single-center study, majority of cases with advanced gastric cancer had a response to Xelox regimen as first line therapy. Xelox regimen had high efficiency with minor toxicities. Overall response rate was 74.99% and nearly 30% of cases had complete response. Our results showed favourable and comparable outcome in comparison to previous study. In the study of Tingsong Yang et al. in 2011,^23^ 75 cases with advanced gastric cancer, who were treated with Xelox regimen had overall response rate of 62.2%; while 4.1% had complete response, 58% partial response and stable disease and progressive disease were 21.6% and 13.5%, respectively. The response rate in Yang and et al. study was lower than our study and complete response differed significantly with our study (4.1% vs. 29%). Median time for progression and median overall survival rate were 5.9 months and 10.8 months. Delay in treatment protocol and dose reduction occurred in 28.3% and 14.6%, which was completely different from our study. Anaemia related to treatment protocol was 62% and neuropathy was 59.5%, which was completely different to our study.^23^ In the study of Dong et al. in 2009, 41 cases with gastric cancer were evaluated.^24^ In this study, 51.2% had response and two cases had complete response. This response rate and complete response were different from our study. Median follow-up time was 9.5 months, median time for progression was 5.6 months and overall survival rate was 9.8 months.^24^ In another study in 2014,^25^ 48 cases with median age of 63.5 years were evaluated. In this study complete response, partial response, stable disease and progressive disease were 4.2%, 44.68%, 36.17%, 14.89 %, respectively.^25^ Response rate was 49.0%. Median time to progression was 10 months and overall survival rate was 29.8 months. The cause of these differences with our study might be related to use of surgery for many cases in the study of Wang.^25^ In another study in 2009 with Mashhadi et al. on advanced gastric cancer, the overall response rate was 60%. They reported 10% complete response and 50% partial response and 10% stable disease. Median survival was 13 months and median event-free survival was 8 months.^26^ In this small study, response rate was acceptable but high grade toxicity was the major problem.

## CONCLUSION

  Our results showed efficacy and tolerability of Xelox regimen for advanced gastric cancer. High percentage of clinical improvement in patients with advanced gastric cancer, out-patient treatment and low incidence of life threatening toxicities revealed that this regiment might be a rational modality for the treatment of gastric cancer. Further larger studies are needed to confirm these data.
